# Medium-Based Noninvasive Preimplantation Genetic Diagnosis for Human α-Thalassemias^-SEA^

**DOI:** 10.1097/MD.0000000000000669

**Published:** 2015-03-27

**Authors:** Haitao Wu, Chenhui Ding, Xiaoting Shen, Jing Wang, Rong Li, Bing Cai, Yanwen Xu, Yiping Zhong, Canquan Zhou

**Affiliations:** From the Reproductive Medicine Center, First Afﬁliated Hospital of Sun Yat-sen University, Guangzhou, Guangdong, China, Guangdong Provincial Key Laboratory of Reproductive Medicine.

## Abstract

To develop a noninvasive medium-based preimplantation genetic diagnosis (PGD) test for α-thalassemias^-SEA^.

The embryos of α-thalassemia^-SEA^ carriers undergoing in vitro fertilization (IVF) were cultured. Single cells were biopsied from blastomeres and subjected to fluorescent gap polymerase chain reaction (PCR) analysis; the spent culture media that contained embryo genomic DNA and corresponding blastocysts as verification were subjected to quantitative-PCR (Q-PCR) detection of α-thalassemia^-SEA^. The diagnosis efficiency and allele dropout (ADO) ratio were calculated, and the cell-free DNA concentration was quantitatively assessed in the culture medium.

The diagnosis efficiency of medium-based α-thalassemias^–SEA^ detection significantly increased compared with that of biopsy-based fluorescent gap PCR analysis (88.6% vs 82.1%, *P* < 0.05). There is no significant difference regarding ADO ratio between them. The optimal time for medium-based α-thalassemias^–SEA^ detection is Day 5 (D5) following IVF.

Medium-based α-thalassemias^–SEA^ detection could represent a novel, quick, and noninvasive approach for carriers to undergo IVF and PGD.

## INTRODUCTION

Thalassemias are the most common monogenic inherited diseases worldwide, and they affect most countries to some extent.^1^ They are prevalent in the Mediterranean area, Middle East, Transcaucasia, Central Asia, Indian subcontinent, and Southeast Asia.^2^ Genetically, thalassemias are a group of autosomal recessive disorders caused by the reduction or absent production of one or more globin chains that constitute hemoglobin (Hb) tetramers.^3^ Two main types, including α- and β-thalassemias, can be distinguished according to the type of globin chain involved. In China, the Southeast Asia deletion (^–SEA^) is the most common homozygous mutation, and the incidence rate ranges from 72.87% to 82.87% in individuals with α-thalassemias.^4^ In many developing countries, prenatal diagnosis is available.^5^ Specifically, prospective screening for α-thalassemias has recently been introduced in several at-risk South Asian countries, such as Hong Kong, Southern China, Thailand, Taiwan, Malaysia, Singapore, the Maldives, and Sri Lanka.^6^

Preimplantation genetic diagnosis (PGD) involves the biopsy of oocyte polar bodies or embryonic cells, and it has become a routine clinical procedure in many in vitro fertilization (IVF) clinics worldwide.^7^ It is extremely helpful for parents who are known carriers of chromosomal abnormalities or single gene disorders because unaffected embryos can be chosen for uterine transfer.^8^ PGD is performed at the following three stages of embryo development: oocyte polar body biopsy before and after fertilization, blastomere biopsy at the cleavage stage, and trophectoderm (TE) tissue biopsy at the blastocyst stage.^9^ Polar body biopsy is not a common practice. A key disadvantage of polar body biopsy is that it cannot assess the paternal genetic contribution; thus, it is not useful for couples who seek to thoroughly select against an allele in the paternal genome.^10^ Additionally, it requires a substantial number of polar bodies for analysis, which is time and resource intensive, especially because some oocytes will not be fertilized and some zygotes will not reach the blastocyst stage; other disadvantages include its high cost, the chance for aneuploidy compensation (2%–4%), and the high risk of aneuploidy (32.5%).^9^ Currently, blastomere biopsy at the cleavage stage is the most common technique. Blastomere biopsy provides sufficient time for genetic diagnosis and the selection of healthy embryos.^11^ However, it also has limitations; up to 60% of embryos at the cleavage stage of development exhibit mosaicism, in which at least one cell has a different ploidy from other cells in the embryo.^12^ In addition, many cleavage stage embryos that are diagnosed with aneuploidy following blastomere biopsy will “self-correct” by the blastocyst stage.^13^ Recently, animal studies have suggested that blastomere biopsy may cause aberrant epigenetic modifications, neurodegenerative disorders, and ovary dysfunction in offspring.^14–16^ Thus, a noninvasive testing method for PGD would be favorable and is an urgently needed option.

In this study, we attempted to establish a novel and noninvasive medium-based testing method to screen for healthy embryos from α-thalassemias^–SEA^ carriers who undergo IVF.

## MATERIALS AND METHODS

### Ethical Approval

Each couple whose embryos were enrolled in the study provided written informed consent. The present study was approved by the Research Ethics Committee of the First Affiliated Hospital of Sun Yat-sen University.

### Patients and Intracytoplasmic Sperm Injection (ICSI) Procedure

All cycles were performed between March 2014 and June 2014 in the First Affiliated Hospital, Sun Yat-sen University. After controlled ovarian stimulation with a long protocol (downregulation using a GnRH agonist and stimulation with recombinant FSH), the couples underwent transvaginal oocyte retrieval and fertilization using ICSI as previously described.^17^ For comparisons of routine and new detection methods, 413 embryos were collected from 76 patients (38 couples) age 20 to 45 years who were carriers of the α-thalassemia^-SEA^ Southeast Asia deletion^-SEA^ genotype. For quantification of the medium-based detection, an additional 148 embryos were obtained from 6 patients who required ICSI.

### Cleavage Stage Biopsy and Medium Collection

After ICSI, all fertilized oocytes were cultured from Day 1 (D1) to D3 in G1 culture medium (Vitrolife, Goteborg, Sweden) and incubated at 37°C under a humidified atmosphere of 5% CO_2_.^18^ Embryos that had six or more cells early on D3 postfertilization were placed in 40 μL of G-MOPS medium (Vitrolife, Goteborg, Sweden) under mineral oil and were biopsied ^19^ following zona ablation using a noncontact laser. One blastomere from each embryo was removed using micromanipulation, washed 3 times with PBS with 1% BSA and placed immediately in RNAse-DNAse-free polymerase chain reaction (PCR) tubes that contained 5 μL of cell lysis with proteinase K (Roche Molecular Biochemicals, Mannheim, Germany). The same quantity of PBS from the third wash was collected as a negative control. The samples were transferred to the Laboratory of Medical Genetics for DNA diagnosis via fluorescent gap PCR analysis of α-thalassemia^-SEA^. The diagnose results were turned out on D5.

After biopsy, the embryos were further cultured in G-2 v5 (Vitrolife, Goteborg, Sweden) until D5 after fertilization. Normal or heterozygous wild-type blastocysts diagnosed by biopsy were transferred to the patient's uterus or cryopreserved for future use.^20^ The affected (homozygous mutant) and undetected embryos, including arrested embryos, were subsequently cultured in 5 μL of G-2 v5 from D5 to D6. To prevent media contamination, individual Pasteur pipettes were used for each embryo. On D6, the blastocysts for final confirmation were transferred to 40 μL of G-MOPS medium (Vitrolife, Goteborg, Sweden) with mineral oil, followed by microscopic zona ablation; they were immediately placed in RNAse-DNAse-free PCR tubes that contained 5 μL of cell lysis with proteinase K for quantitative-PCR (Q-PCR) detection. After the blastocysts were transferred, the culture medium was collected with RNAse-DNAse-free PCR clean tubes and subjected to real-time Q-PCR. The same amount of medium that had not been used to culture embryos was used as negative controls. Appropriate precautions were taken to prevent sample contamination by extraneous cells or DNA. Specifically, the culture embryos, medium collection, and subsequent analytical procedures were performed using sterile, RNAse-DNAse-free PCR clean, LoRetention filter tips with a two-phase filter (Eppendorf, Hamburg, Germany).

### Spent Culture Media Collection for Cell-Free DNA Quantification

Sixty-one oocytes fertilized with ICSI were obtained from additional 6 patients; they were individually cultured from D1 to D3 in G1 culture medium (Vitrolife, Goteborg, Sweden) and individually transferred to 5 μL of G-2 v5 medium on D3. They were transferred to 5 μL of new G-2 v5 medium after one day, and the spent culture media from D3 to D4 and D4 to D5 were collected. The quality of each blastocyst was evaluated by blastocyst scoring system, which well accepted and most widely used in IVF clinic. Embryos with grade ≥3BB serve as suitable candidates for single embryos transfer.^21^ Specifically, 3BB stands for “degree of expansion and hatching status with a full blastocyst with a blastocoel completely filling the embryo; inner cell mass with loosely grouped, several cells; Tropoderm with few cells forming a loose epithelium.”^21^ On D5, 35 derived blastocysts that scored better than 3BB were cryopreserved for future transfer; the remaining 26 blastocysts were transferred to new medium and cultured to D6 prior to cryopreservation. The spent culture media from D5 to D6 were collected and underwent cell-free DNA quantification. Altogether there were 148 (D4, 61+ D5, 61+ D6, 26) samples collected for cell-free DNA quantification.

### Fluorescent Gap PCR Analysis of α-Thalassemia^-SEA^

The protocol used was established in our previous study.^4^ Three α-thalassemia^-SEA^ primers were used in fluorescent gap PCR analysis, which are listed in Table 1. The S1 and S3 primers flank the SEA deletion, whereas the S2 primer anneals within the deleted area. PCR was performed in a 50-μL reaction system that contained 5 μL of lysis of a single biopsied blastomere, 5 μL of DMSO (sigma), 4.5 μL of 10 × PCR neutralizing buffer, 1.5 μL of 25 mmol/L MgCl_2_, 1.5 μL of 10 mmol/l dNTP each, 200 mM each of primer, including S1, S2, and S3, and 0.3 μL of AmpliTap DNA polymerase (ABI) using a Perkin Elmer Cetus 9700 PCR machine. The thermal cycling condition included an initial denaturation at 96°C for 2 minutes, followed by 10 cycles of denaturation at 96°C for 45 seconds, annealing at 57°C for 45 seconds, and elongation at 72°C for 1 minute; 40 cycles of denaturation at 94°C for 45 seconds, annealing at 55°C for 45 seconds, and elongation at 72°C for 1 minute and a final elongation at 72°C for 5 minutes. The PCR products were subsequently analyzed using an ABI 3100 Advant genetic analyzer.

**TABLE 1:**
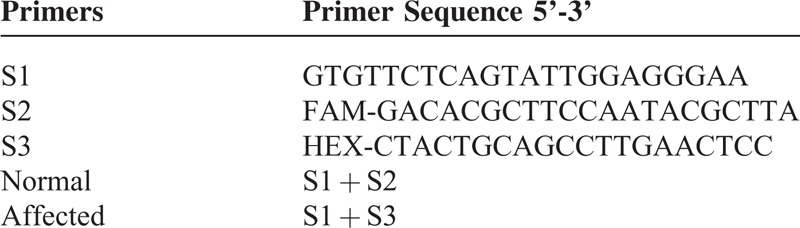
Primer Sets for Fluorescent Gap PCR Analysis

### Real-Time Q-PCR

The procedure was slightly modified according to a previous report.^22^ Highly specific nested PCR was adapted to detect α-thalassemia^-SEA^. Nested PCR involves two sets of primers that are used in two successive runs of PCR; the intention of the second set is to amplify a secondary target within the first run product.^23^ The primers and TaqMan probes are listed in Table 2. The primer for the normal control was designed to avoid locating the common region for the Asian α-thalassemia^-SEA^ (-α4.2 or -α3.7). The reaction conditions were as follows: 1 cycle of 95°C for 10 minutes, followed by 5 cycles of 95°C for 20 seconds and 52°C for 30 seconds, followed by 50 cycles of 94°C for 20 seconds and 60°C for 1 minute. The data were collected at 60°C. For all medium-based detections, 5 μL of medium that had not been used to culture embryos was used as the negative control. For the quantification assay, DNA standards (200, 40, 8, 1.6, and 0.4 pg/μL human genomic DNA, Promega, Madison, WI) were loaded. After subtracting the fluorescence value of the reagent blank (cleavage medium) from each sample, we determined the DNA concentration of the samples from the standard curve by plotting the concentration of DNA standards (mg/mL) (x-axis) against the fluorescence reading (y-axis).^24^

**TABLE 2:**
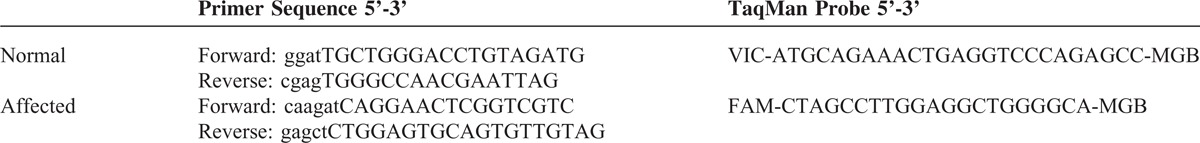
Primer and Probe Sets for Medium-Based Detection

### Statistical Analysis

The data are expressed as the mean ± SD. For outcomes that were measured at a single time point, a two-sample *t* test or analysis of variance (ANOVA) was used to assess differences. Pearson χ^2^ test was applied to analyze the categorical data. Significance was set at *P* < 0.05.

## RESULTS

### Detection of α-Thalassemia^-SEA^ via Fluorescent Gap PCR Analysis

**FIGURE 1:**
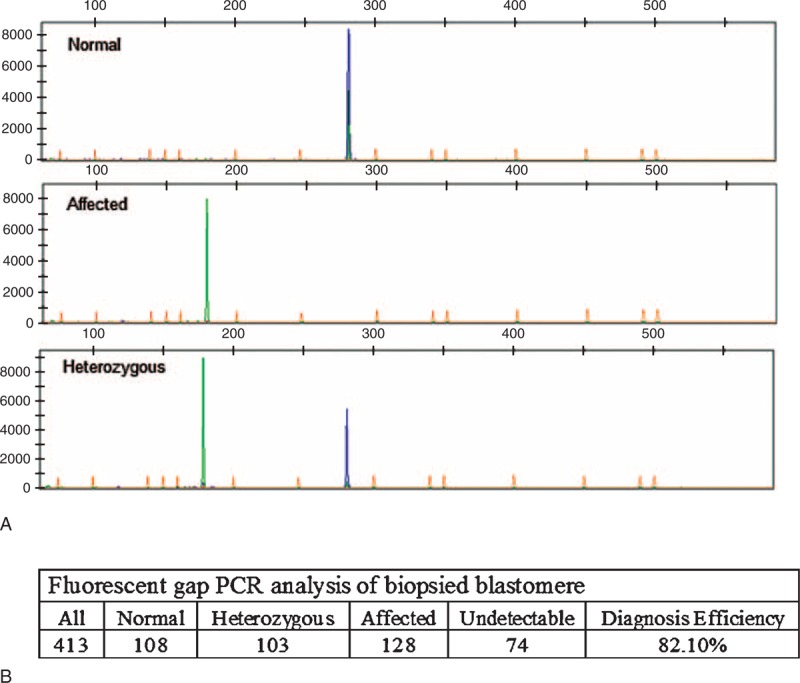
Detection of α-thalassemia^-SEA^ via fluorescent gap PCR analysis. Embryos that had six or more cells early on Day 3 postfertilization were subjected to biopsy and fluorescent gap PCR analysis. **A.** The top, middle, and lower lanes indicate the normal, affected, and heterozygous, respectively. **B.** Results summary.

Four hundred thirteen samples from cleavage-stage biopsies were examined for α-thalassemia^-SEA^ via routine fluorescent gap PCR analysis (Figure 1A). There were 108 normal, 103 heterozygous, and 128 affected (α-thalassemia^-SEA^ deletion) embryos, and 74 samples were undetectable. The ratio for the DNA diagnosis efficiency of α-thalassemia^-SEA^ was 82.1% (339/413, Figure 1B).

### Medium-Based Detection of α-Thalassemia^-SEA^

**FIGURE 2:**
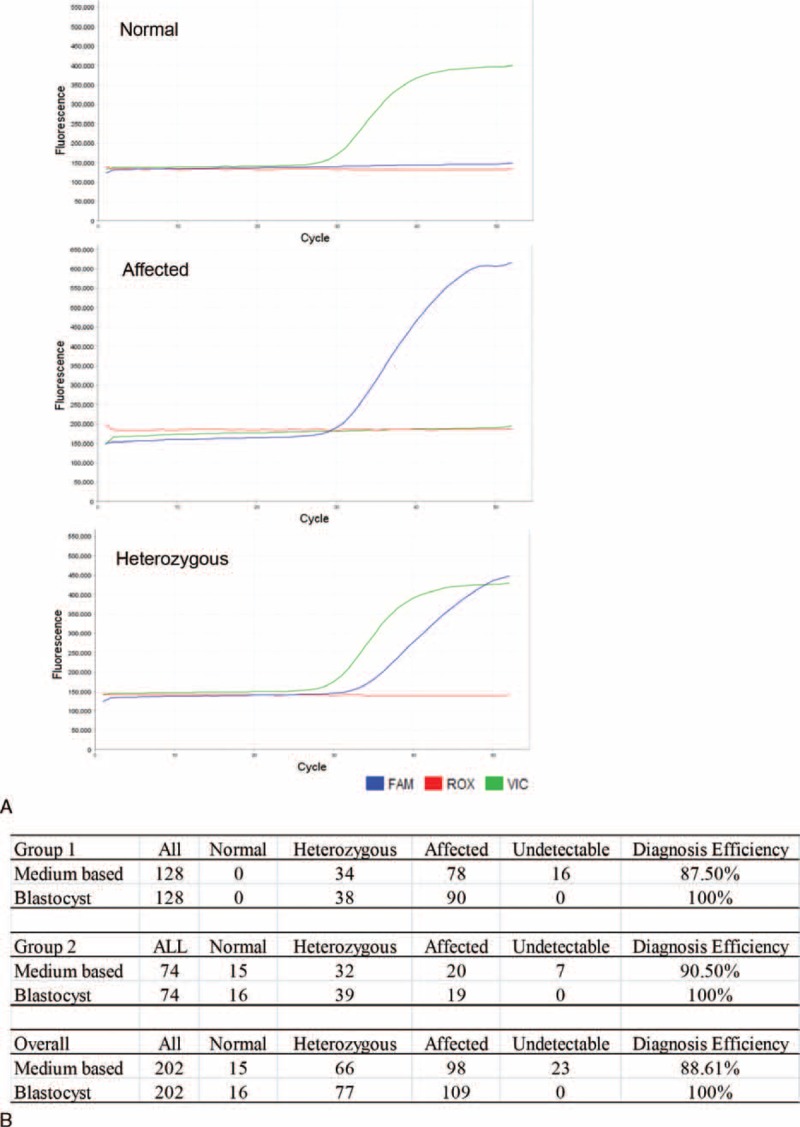
Medium-based detection of α-thalassemia^-SEA^. The affected and undetected embryos determined by biopsy were subsequently cultured in 5 μL G-2 to Day 6. Corresponding medium was collected for detection of α-thalassemia^-SEA^. The blastocysts were lysed for final confirmation. **A.** The top, middle, and lower lanes indicate the normal, affected, and heterozygous, respectively. **B.** Results summary.

As shown in Figure 2A, the VIC S curve along with the flat FAM represents normal Hb (homozygous wild type), whereas the FAM S curve along with the flat VIC represents affected Hb (homozygous mutant). Heterozygous (heterozygous for mutant and wild-type alleles) are shown when both the FAM and VIC indicate S curves.

Two hundred and two samples, including 128 affected (Group 1) and 74 undetectable (Group 2) in fluorescent gap PCR analysis, were subjected to medium-based and blastocyst lysis final detection. The diagnosis efficiency of medium based was 87.5% in Group1 and 90.5% in Group2, with overall 88.6% which was significantly increased compared with that of fluorescent gap PCR (82.1%). The allele dropout (ADO) ratio in heterozygous was 10.5% (4/38) in Group1 and 13.5% (5/37) in Group2 (Figure 2B), with overall 12%. The lysis of the corresponding 202 embryos was examined with Q-PCR for final confirmation as shown in Figure 2B.

### Characterization of Cell-Free DNA

**FIGURE 3:**
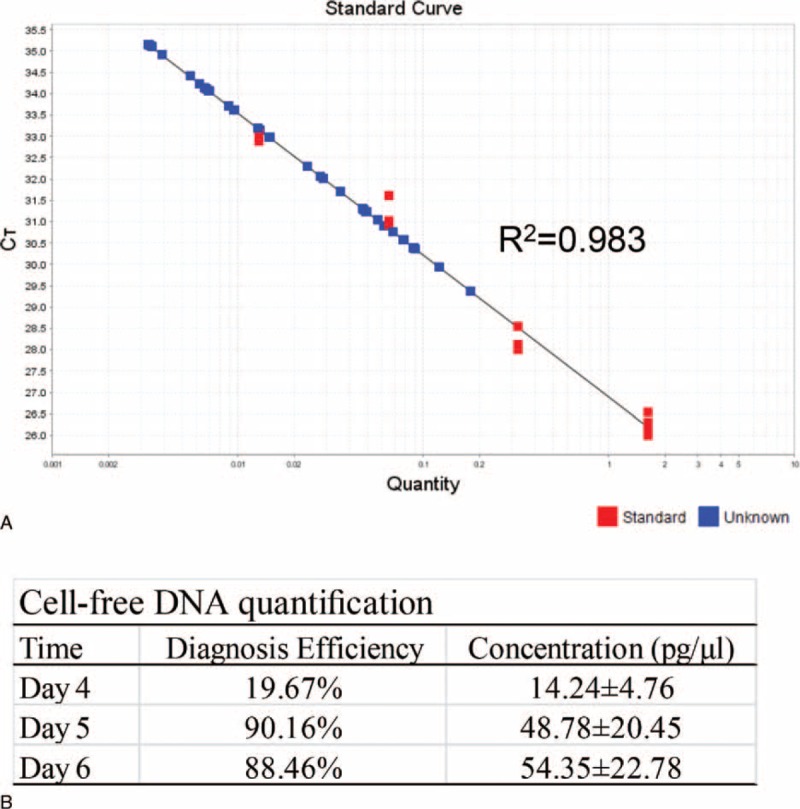
Characterization of cell-free DNA. Another set of integrated embryos without biopsy were subjected to quantification of cell-free DNA via medium-based detection of α-thalassemia^-SEA^. **A.** The standard curved generated by medium-based Q-PCR detection. **B.** Results summary.

To confirm the new method of medium-based detection, an additional 61 integrated embryos that had not been biopsied were subjected to quantification of cell-free DNA in medium. As shown in Figure 3, the detectable ratio of the medium collected at D4 (D3→D4) after fertilization was 19.67% (12/61) with a concentration of 14.24 ± 4.76 pg/μL; this ratio dramatically increased to 90.16% (55/61) with a concentration of 48.78 ± 20.45 pg/μL at D5 and 88.46% (23/26) with a concentration of 54.35 ± 22.78 pg/μL at D6. No significant difference was identified between D5 and D6 (*P* > 0.05).

## DISCUSSION

IVF offers personal approaches for couples according to their specific infertility problems and may include PGD, single-embryo transfer, blastocyst transfer, and optimization of uterine receptivity.^25^ PGD is recommended for couples at risk for specific inherited disorders, such as α-thalassemias^–SEA^ carriers. To the best of our knowledge, this is the first study to introduce a new, noninvasive medium-based testing to screen healthy embryos from α-thalassemias^–SEA^ carriers who undergo IVF.

Noninvasive PGD is attracting increasing attention. First, proteomic technologies and mass spectrometry have indicated that differentially secreted substances could lead to noninvasive viability screening, including chromosomal constitution among preimplantation embryos.^26^ Additionally, time-lapse imaging of embryos has been used as a predictor of good implantation and lower aneuploidy rates among transferable embryos.^27^ Nevertheless, these methods are expensive or time consuming, and they are typically used for embryo and chromosomal evaluation rather than the diagnosis of genetic diseases. Intriguingly, the Palini team sampled blastocoelic fluid from expanded human blastocysts prior to vitrification and subjected the contents to PCR and DNA amplification procedures. The authors confirmed the presence of cell-free genomic DNA in the blastocoelic fluid of approximately 90% of the investigated embryos, and several genes related to the sex of the embryo were identified.^28^ The results from a pilot study also confirmed that testing DNA in blastocyst fluids could have 97.4% and 96.6% concordance with testing the whole embryo for ploidy and TE cell chromosomes, respectively.^29^ In addition, the mitochondrial DNA content in embryo culture medium is significantly associated with human embryo fragmentation, which may provide a novel, noninvasive, and objective tool to grade embryo quality.^24^ In the present study, we utilized, for the first time, an embryo culture medium for the detection of α-thalassemias^–SEA^. The overall diagnosis efficiency significantly increased to 88.6%, compared with 83.7% in the fluorescent gap PCR analysis which is the routine clinical procedure used in many IVF clinics. The latter approach typically requires one or two days to analyze, whereas our method only requires a few hours. For heterozygous embryos, ADO for the normal allele may lead to misdiagnosis as the homozygous mutant embryo and vice versa. The presence of the normal allele in the transferred embryos should offset any adverse consequence because of ADO in the mutant allele in α-thalassemia.^30^ Here, the medium-based overall ADO ratio was 12%, which is not significantly different from a previous report (10.7%, *P* > 0.05).^4^

To further confirm this noninvasive testing and to optimize the procedure, we quantitatively assessed the DNA concentration in the culture medium over time. A previous study demonstrated that the release of nucleic acids by cleaved D2 and D3 human embryos is a common phenomenon because 99% of spent media contained DNA.^24^ To our surprise, the diagnosis efficiency was only 19.67% at D4 after IVF with a cell-free DNA concentration of 14.24 ± 4.76 pg/μL. This inconsistency may be because of the different experimental conditions, in which we excluded the release of DNA from cumulus cells or sperm via extensive isolation and ICSI. In contrast, the diagnosis efficiency increased to 94% and 96% at D5 and D6, respectively. However, no significant difference between D5 and D6 was identified (*P* > 0.05). These data suggest that the optimal opportunity for medium-based α-thalassemias^–SEA^ detection is D5 after IVF.

The advantages of this medium-based detection are obvious. First, in contrast to the routine biopsy analysis, medium-based detection is noninvasive. Second, biopsy may fail as a result of the embryos themselves or by improper sample delivery. Medium-based detection can avoid these situations. Third, biopsy must be performed at a proper point of embryo growth, which requires adequate material prior to the formation of tight connections in the trophoblast.^9^ Furthermore, compared with blastocoelic fluid detection, which is performed at a particular stage of blastocoel formation and requires adequate blastocoelic fluid,^28^ medium-based detection is more flexible; this flexibility makes it suitable to monitor different stages of embryo development because cell-free DNA in medium is stable after D4. Finally, the detection is simple and efficient. The transplantation of fresh healthy embryos can be achieved after 3 hours of medium-based detection.

However, some caveats of our research merit comment. First, we are uncertain as to what extent the sample DNA collected from the culture medium is representative of the embryo because human DNA is often present in embryo-free droplets of protein-supplemented culture medium. However, in our study, no α-thalassemias^–SEA^ were detected in the embryo-free droplets. In addition, the prevention of contamination of the collection medium is critical. Contamination may originate from insufficient washing of the embryos. In our study, we washed off cumulus cells of embryos on D3 thoroughly while they were loose enough. Although there was no detectable contamination in our study, the quality and purity of the collected medium needs further verification, such as the introduction of short tandem repeat (STR) or single-nucleotide polymorphism (SNP) genotyping. Finally, clinic outcome data should be followed to validate this noninvasive screening method.

In summary, we established a novel, quick, and noninvasive method of medium-based α-thalassemias^–SEA^ detection for carriers who undergo IVF and PGD.
